# Mesenteric and Retroperitoneal Mucinous Cystic Neoplasms: A Nongynecologic Process Commonly Managed by Gynecologic Oncologists

**DOI:** 10.1155/crog/7921200

**Published:** 2026-03-02

**Authors:** Judy Hayek, Jennifer Wolf, Alexandra Hamilton, Yong Mei Yin, Margaux J. Kanis

**Affiliations:** ^1^ Division of Gynecologic Oncology, Maimonides/SUNY Downstate Health Sciences University, Brooklyn, New York, USA; ^2^ Department of Obstetrics and Gynecology, NewYork-Presbyterian (NYP) Brooklyn Methodist Hospital, Brooklyn, New York, USA; ^3^ Department of Pathology, NewYork-Presbyterian (NYP) Brooklyn Methodist Hospital, Brooklyn, New York, USA; ^4^ Division of Gynecologic Oncology, NewYork-Presbyterian (NYP) Brooklyn Methodist Hospital, Brooklyn, New York, USA

**Keywords:** diverticulitis, mesenteric–retroperitoneal, mucinous cystic, pelvic mass

## Abstract

Retroperitoneal mucinous cystic neoplasm is a rare pathology. Data regarding diagnosis and treatment are limited and based on case reports and series. We describe a case series of two patients each presenting with a pelvic mass suspicious for an adnexal neoplasm. Both were treated surgically with complete resection of the suspected tumor. Pathology from both specimens revealed mesenteric–retroperitoneal mucinous cystic neoplasm. Uniquely, both patients had diverticulosis and their perioperative course was complicated by diverticulitis. We investigate a possible correlation between diverticular disease and the histopathology of mesenteric retroperitoneal mucinous cystic neoplasms.

## 1. Introduction

Mucinous cystic neoplasms have most frequently been described arising in the pancreas and liver [1–5] with rare case reports of tumors arising in the retroperitoneum or mesentery [6–9]. These tumors are composed of mucinous epithelium and are most often described as low‐grade dysplasia or low malignant potential. They have been described in both males and female patients [5]; in women, they differ from other benign mucinous tumors due to their ovarian‐like stroma [6], but have been described by a variety of names in case reports, limiting comparisons. Of the nonpancreatic and hepatic/biliary reports, most are described as originating from the retroperitoneum, with a few originating from the rectal muscularis propria [9], transverse colon mesentery [6], sigmoid mesentery [8], and periappendiceal mesentery versus mesoappendix [5]. No reports of connections to the bowel lumen or postoperative complications have been described. Here, we describe two cases of mucinous cystic neoplasms arising from the retroperitoneum or mesentery in which the patient′s course was complicated by diverticulitis. All the patients allowed personal data processing, and informed consent was obtained from all individual participants included in the study.

## 2. Case Presentations

### 2.1. Case 1 (1401515970)

An 83‐year‐old who had previously undergone total abdominal hysterectomy 50 years prior for fibroid uterus was sent to the hospital by her primary care physician due to severe hypertension, where she was incidentally found to have a 20‐cm pelvic mass. Bilateral percutaneous nephrostomy tubes were placed due to extrinsic compression of the ureters. She was subsequently referred to gynecologic oncology, and CT of the abdomen and pelvis revealed a large complex midline pelvic mass measuring up to 20.7 cm, thought to be arising from the left ovary, and concerning for cystic ovarian neoplasm (Figure 1). There was enlarged retroperitoneal and pelvic lymphadenopathy, but no ascites or carcinomatosis. She was noted to have partially duplicated urinary collecting systems with moderate bilateral hydroureteronephrosis. On chest CT, she was noted to have a 3.7‐cm mass in the right upper lobe and a 5‐mm nodule in the left upper lobe concerning for lung cancer. Serum tumor markers were collected, with mildly elevated CA 19‐9 (61) and CEA (5.1) but normal CA 125 (22).

Figure 1(a) Case 1: CT abdomen and pelvis of pelvic mass. Complex midline pelvic mass measuring up to 20.7 cm. (b) Case 1: Gross specimen: 18‐cm multiloculated mass.

**Figure:**
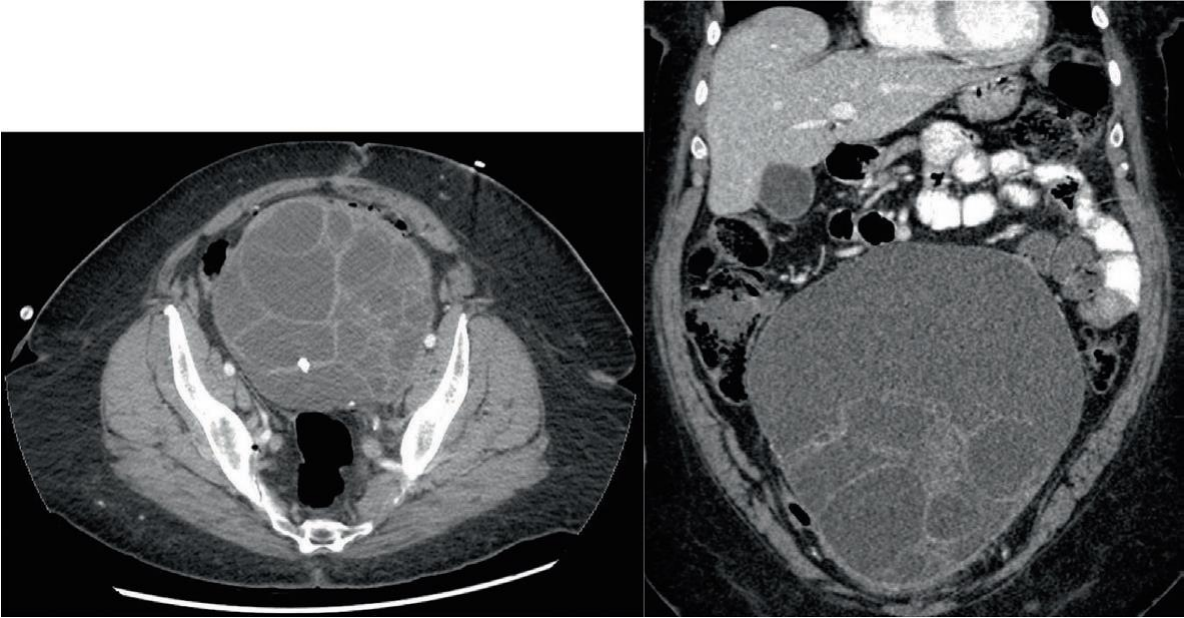
(a)

**Figure:**
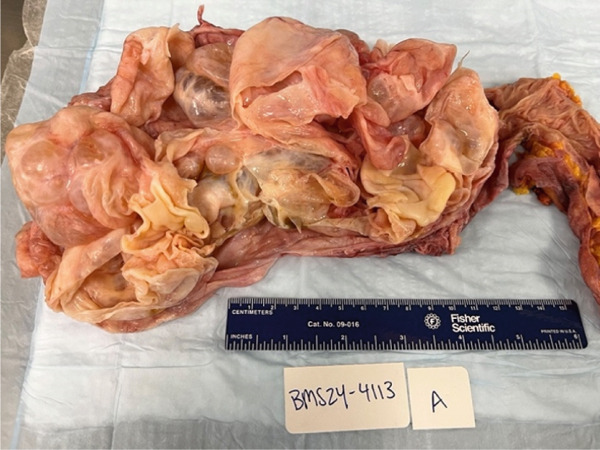
(b)

In a multidisciplinary approach, after discussion with our colorectal surgery colleagues, she was counseled on exploratory laparotomy. The retroperitoneal mass was mobilized and excised, with requirement for small bowel and sigmoid colon resection and reanastomoses due to significant adhesions. Intraoperatively, the pelvic mass was densely adherent to the sigmoid colon and vaginal cuff, but no distinct ovarian tissue was noted, and it was not clearly originating from the infundibulopelvic ligament.

On final pathology, the mass measured 18 cm and was multilocular and cystic. Microscopically, mucinous epithelium was surrounded by ovarian‐like stroma. It was described as mesenteric–retroperitoneal mucinous cystic neoplasm with low‐grade dysplasia. The ovarian‐like stroma was positive for immunohistochemical (IHC) stains CD10, ER, and PR (Figure 2). Both the small bowel and sigmoid colon that were resected were notable for diverticulosis and diverticulitis, but no specimens contained invasive carcinoma.

Figure 2(a) Case 1: Histologic features of the mucinous cystic neoplasm, hematoxylin–eosin stain. Low‐grade mucinous epithelium with ovarian stroma (H&E, 10×). (b) Case 1: Histologic features of the mucinous cystic neoplasm, hematoxylin–eosin stain. Ovarian stroma (ER IHC, 10×).

**Figure:**
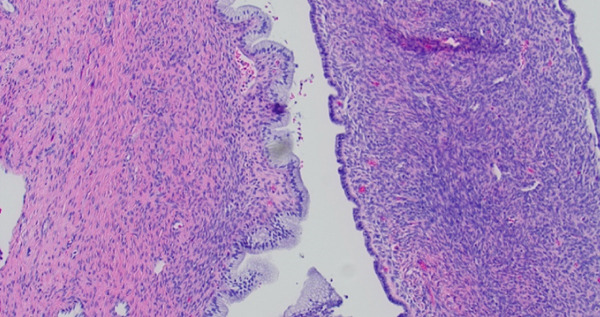
(a)

**Figure:**
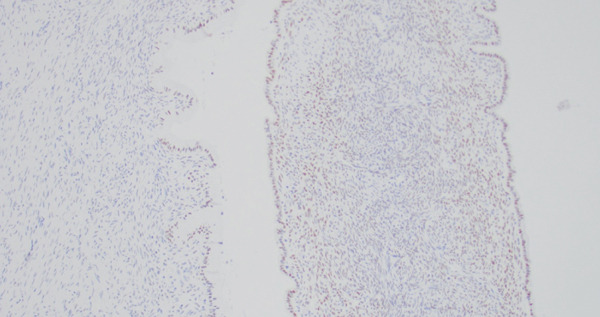
(b)

Postoperatively, she underwent transbronchial lung nodule biopsy and was found to have squamous cell carcinoma of the lung, for which she is currently undergoing treatment.

### 2.2. Case 2 (1002649788)

**Figure 3:**
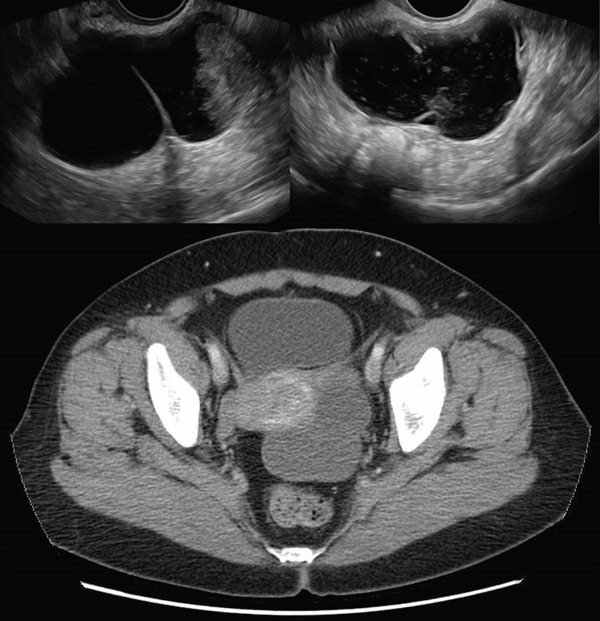
Case 2: Ultrasound and CT abdomen and pelvis of left adnexal mass.

A 63‐year‐old woman presented to the emergency department with left lower quadrant abdominal pain and was found to have a complex ovarian cyst on both transvaginal ultrasound and CT scan. On ultrasound, the mass was described to be within the left adnexa, complex, and cystic, measuring 7.2 x 7.0 x 4.3 cm with a solid component measuring 3.0 cm in greatest dimension. On CT abdomen and pelvis, the left adnexal cystic lesion was measured up to 8.2 cm and the endometrial lining was also noted to be thickened for a postmenopausal woman at 1.6 cm (Figure 3). Additional preoperative workup included collection of serum tumor markers, which were all normal. CA 125 was 8, CEA was 1.3, and CA 19‐9 was 7. The patient had a history of hypertension and diabetes mellitus and no surgical history.

**Figure 4:**
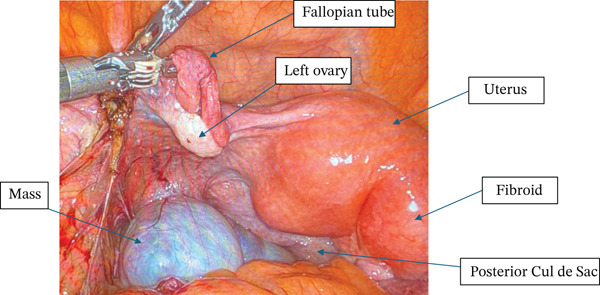
Case 2: Intraoperative finding of cystic mass distinct from the left ovary.

She underwent a robotic‐assisted total laparoscopic hysterectomy, bilateral salpingo–oophorectomy, and excision of the pelvic mass. The bilateral ovaries appeared grossly normal, and the cystic mass was noted to be originating from an approximately 1‐cm thick stalk in the left pelvic sidewall inferior to the adnexa, medial to the ureter and uterine vessels, and distinct from the rectosigmoid colon (Figure 4). On pathology, the bilateral fallopian tubes and ovaries were unremarkable, and there was an endometrial polyp but otherwise the uterus was unremarkable. The pelvic mass measured 8 cm, was unilocular, and noted to be composed of mucinous epithelium surrounded by ovarian‐like stroma. It was described as mesenteric–retroperitoneal mucinous cystic neoplasia with low‐grade dysplasia. The stroma stained positive on IHC for CD10, ER, PR, and WT1 (Figure 5a). The mucinous epithelium stained positive on IHC for CK7, CK20, and CDX2 (Figure 5b). Ki67 was high at 40%–60%; p16 and Napsin A staining were negative; and p53 was nonmutant. These IHC patterns were similar between the first and second case (Table 1).

Figure 5(a) Case 2: Histologic features of mucinous cystic neoplasm, low‐grade mucinous epithelium with ovarian stroma (H&E, 10×). (b) Case 2: Histologic features of mucinous cystic neoplasm, ovarian stroma (ER IHC, 10×).

**Figure:**
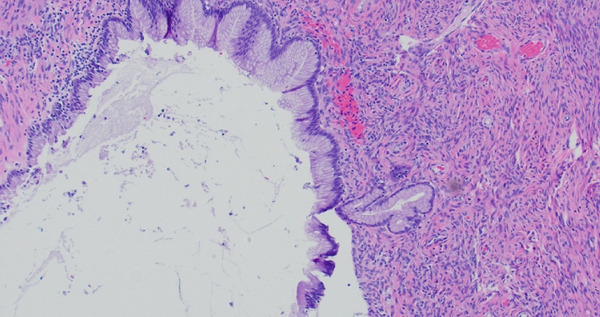
(a)

**Figure:**
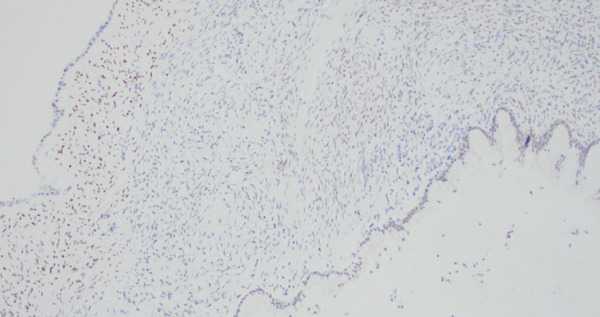
(b)

**Table 1:** Results of immunohistochemistry for the epithelial tissue and stromal cells.

	Case 1	Case 2
Epithelial		
CK7		Positive
CK20		Positive
CDX2		Positive
Napsin A		Negative
P53		Nonmutant
P16		Negative

Stromal		
CD10	Positive	Positive
ER/PR	Positive	Positive
WT1		Positive

The patient was discharged on the day of surgery, but the postoperative course was complicated by her return to the emergency department due to diarrhea and back pain. On CT abdomen and pelvis, there was not a vaginal cuff abscess, but she was noted to have focal bowel wall thickening involving the distal descending and proximal sigmoid colon with pericolonic inflammation. She was started on a course of antibiotics for acute diverticulitis, and colonoscopy revealed multiple small and large‐mouthed diverticula in the left colon.

## 3. Discussion

Most pelvic cystic masses in women arise from the ovaries. Mucinous cystic neoplasms have most frequently been described arising in the pancreas and liver and rarely retroperitoneal or mesenteric [1–4]. They can easily be confused for ovarian neoplasms on initial presentation and can present as benign, borderline, or malignant; benign lesions being the rarest of all [10].

The etiology of retroperitoneal mucinous cystic lesions is unknown given their rarity. Available data mainly relies on case series and reports. One of its kind, a meta‐analysis by Wolf et al. described 144 cases over a duration of 40 years, the majority of which were malignant. They found no preoperative factors predicting tumor nature [11]. Most of these tumors are found incidentally, and if symptomatic, it is commonly due to their large size. It is reported that they present in middle‐aged women but have been described in men as well [12, 13].

The exact histopathology of these tumors has not been determined. Some hypothesized origins include heterotopic ovarian tissue, mullerian remnant, mucinous metaplasia of retroperitoneal mesothelium, mesonephric remnant, or enteral origin from colonic duplication [14]. On a microscopic level, these tumors, as seen in our cases, tend to have mucinous epithelium that is surrounded by ER/PR positive ovarian‐like stroma, which supports the first two of the abovementioned theories. Tomisaki et al. described that female hormones may contribute to the development and growth based on these theories [13]. The presentation of mucinous cystic neoplasms in our postmenopausal patients disagrees with this argument.

The most recently accepted hypothesis has been the mucinous metaplasia of coelomic epithelial remnant; [13] it is thought that during embryology, the coelomic epithelial cells invaginate and are trapped in the retroperitoneum. These cells then transform into mucinous epithelium that later transforms into mucinous cystic neoplasms, a theory that resonates in the gynecologic world as a proposed pathohistological process for endometriosis.

Similarly, in the gastrointestinal field, there has been a proposed correlation between diverticulitis and metaplasia. It is suggested that chronic inflammation in diverticula could result in metaplasia and potentially cancerous changes [15, 16]. Despite the lack of data supporting diverticulitis as an instigator for retroperitoneal mucinous cystic neoplasms, our two cases being mesenteric–retroperitoneal suggest a potential correlation, but further data is needed to support this theory.

The patient in Case 2 underwent colonoscopy which ruled out underlying malignancy; however, in the literature, a malignant transformation of a benign mesenteric mucinous cyst has been described. Bury and Pranicolo described a case of malignant tumor in a recurrence after incomplete excision of a benign mesenteric retroperitoneal mucinous cystic neoplasm [17]. Banerjee et al. described a patient with a borderline mesenteric tumor that later recurred as metastatic disease to the mediastinum [18].

Since there is no tool for diagnosis preoperatively, definitive diagnosis remains postoperative relying on pathologic examination. In terms of management, given its rarity, there are no set guidelines to follow. Nevertheless, based on our literature review, the consensus is that complete excision is advised to decrease the risk of recurrence or malignant transformation.

In conclusion, benign retroperitoneal mucinous cystic neoplasms are rare, and management relies on published case reports. We describe two cases of mucinous cystic neoplasms arising from the mesenteric–retroperitoneum in which the patients′ courses were complicated by diverticulitis. We suggest a potential correlation between the inflammatory activity of diverticulitis inciting a metaplastic process leading to the formation of these tumors. Data on this theory are scarce and more research is warranted.

## Funding

No funding was needed for this study.

## Conflicts of Interest

The authors declare no conflicts of interest.

## Data Availability

The data that support the findings of this study are available on request from the corresponding author. The data are not publicly available due to privacy or ethical restrictions.
